# The influence of fluid resuscitation strategy on outcomes from dengue shock syndrome: a review of the management of 691 children in 7 Southeast Asian hospitals

**DOI:** 10.1136/bmjgh-2024-017538

**Published:** 2025-03-11

**Authors:** Huynh Trung Trieu, Nguyen Lam Vuong, Nguyen Thanh Hung, Tuan Nguyen Minh, Vinh Chau Nguyen Van, Tu Qui Phan, Truong An Nguyen, Su Nguyen Thi Minh, An Nguyen Thi Truong, Em Jun Min, Heng Kai Voon, Shirley Chan Huey Ling, Hue Yuen Ling, Lim Eng Seng, Lucy Lum Chai See, Sharifah Faridah Syed Omar, Amnasewary Ramakrishnan, Aiu Jer Ling, Alia Zubaidah Bahtar, Nachal Nachiappan, Kyaw Zin Wai, Kyi San Thi, Yee Mon Lwin, Nick Ward, Anushka Ward, Sophie Yacoub, Hung Trinh, Phung Khanh Lam, Bridget Wills

**Affiliations:** 1 Hospital for Tropical Diseases, Ho Chi Minh City, Viet Nam; 2 Oxford University Clinical Research Unit, Ho Chi Minh City, Viet Nam; 3 Dengue, Oxford University Clinical Research Unit, Ho Chi Minh City, Viet Nam; 4 University of Medicine and Pharmacy, Ho Chi Minh City, Viet Nam; 5 Children’s Hospital 1, Ho Chi Minh City, Viet Nam; 6 Children’s Hospital, Ho Chi Minh City, Viet Nam; 7 Hospital for Tropical Diseases, Ho Chi Minh, Viet Nam; 8 University of Malaya Medical Center, Kuala Lumpur, Malaysia; 9 Hospital Ampang, Ampang, Selangor, Malaysia; 10 Hospital Kajang, Kajang, Selangor, Malaysia; 11 Hospital Tengku Ampuan Rahima, Klang, Malaysia; 12 Yankin Children’s Hospital, Yangon, Myanmar; 13 Royal College of Paediatrics and Child Health, London, UK; 14 Nuffield Department of Medicine, University of Oxford, Oxford, UK

**Keywords:** Dengue, Treatment, Arboviruses

## Abstract

**Introduction:**

The pathognomonic feature of dengue shock syndrome (DSS) is a transient capillary leak syndrome resulting in profound intravascular volume depletion. WHO management guidelines recommend particular parenteral fluid regimens during the critical leakage phase, including synthetic colloid solutions in certain circumstances. We set out to describe the actual fluid management strategies employed in different settings and to investigate relationships with clinical outcomes.

**Methods:**

We performed a retrospective review of paediatric DSS cases managed at seven hospitals across Malaysia, Myanmar and Vietnam. We explored the effects of both initial resuscitation (crystalloid alone or mixed crystalloid/colloid in the first 2 hours) and general management: group 1 (conservative-colloid, crystalloid only), group 2 (intermediate-colloid, colloid for 1–4 hours) or group 3 (liberal-colloid, continuous colloid for more than 4 hours) categorised according to the fluid given over the first 6 hours in clinically stable patients. We incorporated an inverse probability weighting score to adjust for potential differences in baseline severity.

**Results:**

Among all 691 patients, respiratory compromise (HR 2.08, p=0.022), requirement for nasal continuous positive airway pressure (NCPAP)/ventilation (OR 2.34, p<0.045) and days in hospital after DSS onset (risk ratio, RR 1.33, p=0.032) were significantly worse for mixed crystalloid/colloid versus crystalloid-only initial resuscitation regimens, after adjusting for baseline severity. Among the 547/691 children who stabilised within 2 hours, although a liberal-colloid general management strategy (group 3) was associated with a reduction in recurrent shock episodes (RR 0.13, p=0.043) when compared with a conservative-colloid strategy (group 1), the risks for respiratory compromise (OR 8.84, p<0.001) and requirement for NCPAP/ventilation (OR 8.16, p<0.001) were markedly increased. Additionally, the respective costs for group 3 vs group 1 were significantly higher.

**Conclusions:**

The study highlights the potential benefits and risks of using colloid solutions in children with DSS. Formal randomised trials could help determine the most effective and safe parenteral fluid regimens for paediatric DSS. In the meantime, prolonged use of colloid solutions may be inappropriate, especially in settings without access to respiratory support.

**WHAT IS ALREADY KNOWN ON THIS TOPIC**

- There are few clinical trials on the influence of different fluid management strategies on dengue shock syndrome (DSS) outcomes. The largest of three single-centre, randomised double-blind trials established that a balanced crystalloid solution was as effective as synthetic colloids for the initial resuscitation of children with moderately severe DSS.

**WHAT THIS STUDY ADDS**

- Incorporating colloids into the initial resuscitation strategy did not provide better outcomes than use of crystalloids alone. Shock recurred less frequently in those managed subsequently with a liberal-colloid approach, but the risk for respiratory compromise was markedly increased.

**HOW THIS STUDY MIGHT AFFECT RESEARCH, PRACTICE OR POLICY**

- Administration of synthetic colloids remains an essential element of the current WHO guidelines for paediatric DSS, but liberal-colloid use should not be encouraged.

## Introduction

 Dengue is a mosquitoborne viral infection of major global significance. It is endemic in more than 100 countries and is rapidly spreading to new geographical areas, with over half of the world’s population now at risk.^1^ An estimated 390 million infections occur annually worldwide, among which around 25% are symptomatic.^2^ Most symptomatic individuals experience a self-limited influenza-like febrile episode lasting 3–7 days, but in a small proportion of cases, potentially life-threatening complications develop. The most notable complication, particularly prevalent in children and young adults, is a transient systemic vascular leak syndrome that can progress to hypovolaemic shock (dengue shock syndrome, DSS) if severe.^3^ Thrombocytopaenia and a characteristic coagulopathy are almost invariably present, typically associated with only minor clinical bleeding. However, severe haemorrhage can occur, usually in association with profound shock.^4^

Since no disease-specific therapeutics are available, successful management of DSS relies on good supportive care, primarily judicious use of intravenous fluids to restore and maintain adequate intravascular volume. However, meticulous attention to detail is necessary to limit iatrogenic complications, in particular development of respiratory compromise due to volume overload. After initial resuscitation and cardiovascular stabilisation, significant volume depletion may recur during the critical 24–48 hours when vascular permeability remains increased, potentially resulting in recurrent episodes of cardiovascular compromise, often termed ‘reshock’. Some 20%–30% of children with DSS develop reshock, among whom about 30% will have repeated episodes, substantially increasing the risk of death.^5^,^8^ The WHO dengue management guidelines recommend prompt infusion of a 10–20 mL/kg crystalloid bolus for children with established DSS, followed by a tapering crystalloid fluid regimen that can be supplemented by boluses of synthetic colloid solutions if reshock occurs.^3^ If managed in dedicated high dependency/intensive care unit (HDU/ICU) environments by experienced staff, mortality rates below 1% are achievable.^6^ However, in dengue-endemic countries, DSS case numbers are high, and HDU/ICU facilities with the capacity to provide respiratory support are scarce, and the economic and resource burden for local health services is often considerable.^9^

The evidence base for the current WHO guidelines is limited, with the recommendations based primarily on expert opinion. In 2025, following on from an initial pilot and a small trial to explore the feasibility of conducting dengue clinical trials in Vietnam, a randomised blinded intervention trial established that a balanced crystalloid solution was as effective as either a dextran or a starch-based colloid for initial resuscitation of children with moderately severe DSS.^10^ However, no research has been carried out to formally evaluate the effectiveness of the overall WHO fluid management guidelines, or to investigate optimal regimens for decompensated or recurrent shock, specifically the recommendation to use synthetic colloid solutions. Following a series of major publications highlighting potential adverse events associated with their use, administration of synthetic colloids for volume expansion has become controversial in high-income settings.^11 12^ Although there are no reports specific to the management of DSS, in other situations when isotonic crystalloids alone are inadequate for resuscitation of hypotensive shock, albumin solutions are now the preferred choice, often combined with inotropic agents.^13^ However, in low-income and middle-income country (LMIC) settings, where albumin solutions are prohibitively expensive, synthetic colloids continue to be widely deployed as rescue therapy for DSS, in the belief that their use reduces the overall volume of fluid used and thus minimises the need for respiratory support. Many countries have developed recommendations that are loosely based on the WHO guidelines but vary considerably in the specific details (type of fluid, bolus volume, duration) of the regimens proposed.^14^,^17^ In particular, prolonged colloid therapy for reshock (4–6 hours vs 1–2 hours) is becoming established in some places,^18^ potentially increasing the risk for adverse effects.

In this study, we aimed to describe the fluid management strategies actually used for DSS at seven centres of excellence across Southeast Asia and to investigate potential effects of these strategies on major outcomes.

## Methods

### Study design

The primary inclusion criterion for the study was having a clinical diagnosis of DSS on discharge from designated wards at selected centres of excellence across Malaysia (four sites), Myanmar (one site) and Vietnam (two sites). We included all sequential cases without specific age limits. In hospitals with a very high cases burden, we limited the case selection to one or two specialist dengue wards plus the HDU/ICU. Potential cases were identified from the central electronic hospital register, or from direct examination of relevant ward admission/discharge record books. The in-patient records of all potential cases were subsequently reviewed by one of the study clinicians to confirm the discharge diagnosis.

A unique case report form (CRF) was used to extract the following information from all included cases: clinical history and baseline severity indicators at presentation with DSS; all therapeutic interventions throughout the hospital course, in particular detailed information on all intravenous fluids given (timing, type, rate of administration, etc); all cardiovascular observations recorded from onset of DSS for 72 hours or until discharge/death (in most centres pulse and blood pressure were measured every 1–2 hours until stable, then with gradually reducing frequency over 24 hours); adverse events including occurrence and timing of reshock episodes, development and severity of bleeding, respiratory distress, organ impairment etc; and final outcome. A unique code number was assigned to each record to maintain anonymity. In each country, a small team of dengue-experienced doctors and nurses were trained to extract the information, closely supervised by the core Vietnamese study team. CRFs were subsequently transferred to HCMC for double data entry into a secure electronic database designed to maintain patient and site anonymity.

### Classification of fluid management strategies

Each hospital had their own general guidelines for immediate (1–2 hours) DSS management, typically informed by a rapid severity assessment performed by junior medical staff. Subsequent management was determined by the response to the initial fluid resuscitation, usually in consultation with senior staff. Different treatment patterns were apparent at the different centres, particularly in the preference for early versus late intervention with colloids, and the length of time that colloids were given after achieving cardiovascular stability.

To gain insight into possible effects of different attitudes towards fluid management on outcomes, we classified the strategies applied for each individual in two ways (see online supplemental appendixx for more details): (a) Initial resuscitation: either crystalloid alone or mixed crystalloid and colloid fluids, given during the first 2 hours after presentation with DSS and (b) General management strategy: among patients who improved within 2 hours and remained cardiovascularly stable for at least the remainder of the first 6 hours, we categorised the fluid therapy given within this 6-hour window as follows: conservative-colloid (group 1)—received crystalloid only at this time; intermediate-colloid (group 2)—received crystalloid plus a colloid solution for 1–4 hours and liberal-colloid (group 3)—continuous use of a colloid solution for more than 4 hours. We used these three groupings to infer the physicians’ attitudes towards continuing use of colloid in patients who were apparently cardiovascularly stable after initial resuscitation.

### Outcome definitions

The main outcomes of interest were occurrence of reshock and development of respiratory distress. Only events occurring more than 6 hours after recovery from the initial episode of shock were included—that is, after the time window used to define the fluid management strategies employed for each individual when apparently stable. We also assessed the time to initial shock recovery, the number and timing of reshock episodes, and the time to final shock recovery—that is, the time to achieve stable haemodynamic status (no further reshock episodes) while receiving no/maintenance only parenteral fluid and without inotropic support. Formal definitions for all outcomes, all of which were derived by applying algorithms to the electronic dataset, are presented in online supplemental appendix S1.

### Statistical analysis

Having extracted the data from the hospital files of all selected cases, for this analysis, we elected to exclude individuals aged more than 16 years (to maintain comparability between sites, since different age-related ward admission policies were in operation), and also those referred from other hospitals after commencing resuscitation (since we could not access data describing the fluid administered up to the point of transfer). Descriptive statistics are presented as median and IQR for numeric variables, and frequency and percentage for categorical variables.

We used Cox proportional hazard models to compare the time-to-event outcomes across the various fluid strategy groups—time to initial shock recovery, time to first reshock episode and time to final shock recovery. For binary outcomes such as development of respiratory distress, severe bleeding and liver impairment, we used logistic regression models, while negative binomial regression models were used for numeric outcomes such as number of reshock episodes and length of hospital stay. HRs, ORs and risk ratios (RRs), together with their 95% CIs, are reported for the time-to-event, binary and numeric outcomes, respectively.

In recognition of the fact that the patient’s initial severity at presentation with DSS is highly likely to influence management decisions, we developed an inverse probability weighting (IPW) score to adjust for the potential lack of equivalence between patients. This type of weighting can reduce confounding and some forms of selection bias.^19^ For the initial resuscitation strategy, we used a logistic regression model to calculate the IPW, incorporating variables that are recognised in the international literature as indicators of DSS severity^3^; the nine input variables we included were age and body weight, together with day of illness, heart rate, systolic blood pressure, pulse pressure, haematocrit level, platelet count and respiratory distress, all assessed at onset of DSS. For the general management strategy, we used all the previous variables plus four additional variables (maximum heart rate, minimum systolic blood pressure, minimum pulse pressure and maximum haematocrit within the first 6 hours) to calculate the IPW. SEs were calculated using a robust sandwich-type variance estimator. All cases with missing data for any of the variables selected for the IPW calculations were excluded from the adjusted analyses.

All the analyses were performed with R V.3.4.1. The IPW calculations were done with the ‘ipw’ package.^19^

### Economic evaluation

We also assessed the relative cost of different fluid management strategies, focusing specifically on Vietnam. For each patient, we included expenditure for bed costs, routine laboratory tests, intravenous fluids, blood products, respiratory support and antibiotic therapy, as described in online supplemental appendix S2.

### Patient and public involvement

Patients or the public were not involved in the design, conduct, reporting or dissemination plans of our research.

## Results

Data were extracted from a total of 915 files among the 927 potential DSS cases identified from the initial screen of hospital discharge records for dengue seasons across 2015–2017 (online supplemental figure S1). As noted above, for this analysis, we focused on children with DSS managed throughout at the same facility, excluding individuals aged over 16 years (54 patients), referred cases (163 patients) and patients for whom data were incomplete or conflicting (7 cases). Thus, the patient population used for the analysis of initial resuscitation strategy comprised 691 cases, 572 of whom received crystalloid only for the first 2 hours while 119 received mixed crystalloid/colloid fluid during this period. Among these 572 cases, 79 did not achieve cardiovascular stability within 2 hours and 65 decompensated again within the first 6 hours, leaving 547 children in the analysis population for evaluation of general management strategy. Of these patients, 418 were classified in group 1 (conservative-colloid), 45 in group 2 (intermediate-colloid) and 84 in group 3 (liberal-colloid). In total, 11 children died; all 11 were included in the initial resuscitation analysis population, but only 3 were stable and eligible for inclusion in the general management analysis population at 6 hours.

Data quality was generally good (online supplemental appendix S3, online supplemental figure S2-S4), with information available for most parameters at presentation with DSS, apart from respiratory status and confirmatory dengue diagnostic testing. Pulse and blood pressure monitoring was frequent initially, and these parameters were recorded at least every 2 hours in 80% of cases after the first 6 hours. The amount of missing data for most of the defined outcomes was also small, apart from severe liver impairment and acute kidney injury.

Baseline characteristics at onset of DSS are presented in table 1. The median age across the different groups included in the various analyses was 8–9 years, with similar proportions of males and females; in total, 39 children below 1 year of age were included, with representation across all three countries. Most children developed shock on day 5 of illness. In 102/689 (15%), there were signs of profound volume depletion (pulse pressure ≤10 mm Hg)^8^ at presentation, but respiratory distress was noted in only 9/643 (1%) of cases with information at this time. The various cardiovascular and laboratory parameters were generally slightly more deranged in those who subsequently received a colloid for resuscitation. In 91/691 (13%) patients, some parenteral fluid had been given before onset of shock, with a higher proportion of subsequent colloid recipients receiving preshock fluid (for initial resuscitation, 63/572 (11%) crystalloid-only recipients vs 28/119 (24%) mixed fluid recipients). Among the 371 cases in whom dengue diagnostic tests were performed, 347 (94%) were positive.

**Table 1:** Baseline characteristics recorded at onset of DSS and summary of overall clinical outcomes across the two fluid strategy analyses

	Initial resuscitation				General management strategy					
	n	Crystalloid only (N=572)	n	Mixed fluid (N=119)	n	Conservative-colloid(group 1, N=418)	n	Intermediate-colloid (group 2, N=45)	n	Liberal-colloid (group 3, N=84)
Baseline characteristics										
Gender male	572	286 (50)	119	62 (52)	418	210 (50)	45	21 (47)	84	52 (62)
Age (years)	572	9 (5; 11)	119	8 (5; 10)	418	9 (6; 12)	45	9 (7; 11)	84	8 (4; 10)
Admission from home	572	521 (91)	118	98 (83)	418	389 (93)	45	38 (84)	84	64 (76)
Weight (kg)	572	27 (18; 40)	119	25 (16; 36)	418	29 (19; 41)	45	28 (20; 38)	84	26 (16; 37)
Illness day	572	5 (5; 6)	119	5 (5; 6)	418	5 (5; 6)	45	5 (5; 6)	84	5 (5; 6)
Pulse rate (per minute)	572	120 (107; 130)	119	122 (116; 140)	418	120 (105; 130)	45	120 (110; 136)	84	121 (114; 144)
Systolic blood pressure (mm Hg)	571	90 (85; 100)	118	90 (80; 100)	417	92 (90; 100)	45	90 (80; 100)	83	90 (80; 100)
Pulse pressure	571		118		417		45		83	
≤10 mm Hg		54 (9)		48 (41)		29 (7)		25 (56)		16 (19)
>10 mm Hg		517 (91)		70 (59)		388 (93)		20 (44)		67 (81)
Respiratory rate (per minute)	441	25 (22; 28)	103	28 (24; 30)	331	24 (22; 28)	39	24 (20; 28)	79	26 (23; 30)
Respiratory distress	532	5 (1)	111	4 (4)	400	1 (0)	42	1 (2)	82	4 (5)
Haematocrit (%)	570	47 (44; 50)	118	49 (45; 52)	417	47 (44; 50)	45	49 (46; 52)	84	46 (44; 51)
Platelet count (K/uL)	540	34 (19; 53)	118	26 (18; 44)	390	34 (19; 55)	45	25 (19; 44)	84	28 (18; 48)
Dengue test positive	286	268 (94)	85	79 (93)	191	176 (92)	24	23 (96)	52	49 (94)
Received fluid before shock	572	63 (11)	119	28 (24)	418	42 (10)	45	7 (16)	84	19 (23)
Type of preshock fluid	58		28		35		8		15	
Crystalloid only		57 (98)		24 (86)		34 (97)		8 (100)		13 (87)
Crystalloid and colloid		1 (2)		4 (14)		1 (3)		0 (0)		2 (13)
Volume of preshock fluid (mL/kg)	58	13 (4; 40)	28	20 (5; 66)	35	19 (5; 39)	8	13 (4; 46)	15	10 (4; 61)
Clinical outcomes										
Fluid given in the first 24 hours (mL/kg)	572	118 (85; 131)	119	123 (96; 143)	418	118 (85; 127)	45	119 (98; 137)	84	136 (119; 146)
Crystalloids	572	97 (72; 118)	116	50 (25; 70)	418	114 (80; 120)	45	70 (61; 82)	82	38 (20; 50)
Colloids	182	43 (24; 75)	119	69 (35; 107)	83	32 (14; 51)	45	35 (30; 65)	84	95 (76; 110)
Fluid given in the first 72 hours (mL/kg)	572	127 (105; 154)	119	140 (112; 175)	418	125 (105; 143)	45	145 (121; 174)	84	157 (135; 189)
Crystalloids	572	116 (84; 130)	116	61 (27; 88)	418	120 (98; 131)	45	90 (80; 106)	82	40 (20; 55)
Colloids	192	55 (28; 99)	119	70 (35; 122)	89	48 (21; 71)	45	36 (30; 70)	84	112 (83; 140)
Reshock	572	83 (15)	119	25 (21)	418	53 (13)	45	16 (36)	84	1 (1)
Time to first reshock episode (hours)	83	14 (9; 18)	25	10 (8; 15)	53	12 (9; 16)	16	12 (10; 17)	1	16 (16; 16)
Number of reshock episodes	83		25		53		16		1	
1		66 (80)		18 (72)		43 (81)		11 (69)		1 (100)
2		15 (18)		7 (28)		9 (17)		5 (31)		0 (0)
3		2 (2)		0 (0)		1 (2)		0 (0)		0 (0)
Respiratory compromise	572	87 (15)	119	53 (45)	418	33 (8)	45	9 (20)	84	52 (62)
Time to respiratory compromise (hours)	87	22 (10; 31)	53	10 (4; 20)	33	21 (10; 27)	9	18 (11; 26)	52	11 (4; 22)
Requirement for NCPAP	572	24 (4)	119	26 (22)	418	10 (2)	45	2 (4)	84	29 (35)
Requirement for ventilation	572	11 (2)	119	9 (8)	418	4 (1)	45	0 (0)	84	4 (5)
Treated with a diuretic	572	53 (9)	119	35 (29)	418	20 (5)	45	4 (9)	84	40 (48)
Treated with an inotrope	572	13 (2)	119	10 (8)	418	7 (2)	45	0 (0)	84	5 (6)
Time to inotrope use (hours)	13	20 (8; 42)	10	17 (11; 28)	7	26 (14; 52)	0	NA	5	17 (14; 29)
Duration of inotrope use (hours)	13	8 (0; 40)	10	106 (20; 200)	7	4 (1; 29)	0	NA	5	185 (27; 186)
Severe bleeding*	572	22 (4)	119	8 (7)	418	9 (2)	45	2 (4)	84	4 (5)
Severe liver impairment†	224	19 (8)	80	9 (11)	150	7 (5)	29	4 (14)	76	6 (8)
Acute kidney injury†	192	6 (3)	72	1 (1)	128	2 (2)	20	0 (0)	76	1 (1)
Time to initial shock recovery (hours)	572	1 (1; 2)	119	1 (1; 2)	418	1 (0; 1)	45	1 (1; 2)	84	1 (0; 1)
Final status	572		119		418		45		84	
Recovered (final SRT known)		555 (97)		107 (90)		411 (98)		44 (98)		78 (93)
Recovered (final SRT unknown)		8 (1)		10 (8)		4 (1)		1 (2)		6 (7)
Time to final shock recovery (hours)†	555	11 (9; 21)	107	19 (10; 28)	411	10 (9; 17)	44	23 (11; 29)	78	15 (10; 26)
Death		9 (2)		2 (2)		3 (1)		0 (0)		0 (0)
Days in hospital after DSS onset	572	4 (3; 5)	119	5 (4; 6)	418	4 (3; 5)	45	4 (4; 5)	84	5 (4; 6)

Summary statistic is absolute count (%) for categorical variables and median (interquartile rangeIQR) for continuous data.

* One patient in the crystalloid-only group had severe GI bleeding at presentation with shock. One patient in the mixed-fluid group had severe epistaxis at presentation with shock and required nasal packing. In the remaining cases, severe bleeding occurred after shock resuscitation had commenced.

† Patients with at least one relevant laboratory test (liver transaminase or creatinine) performed at shock presentation or during DSS treatment are included here; in the majority with abnormalities, these were already present on the tests performed at presentation (see online supplemental appendixappendix).

‡ For the crystalloid -only/conservative-colloid groups, this refers to use of a colloid solution after the 2hr hours/6hr hours window that was used to define the relevant management strategy.

§ In addition to the 11 deaths, there were 18 survivors for whom final shock recovery time could not be defined (see online supplemental appendix S3appendix S3 for details).

DSS, dengue shock syndrome; NCPAP, nasal continuous positive airway pressure; SRT, shock recovery time

**Figure 1:**
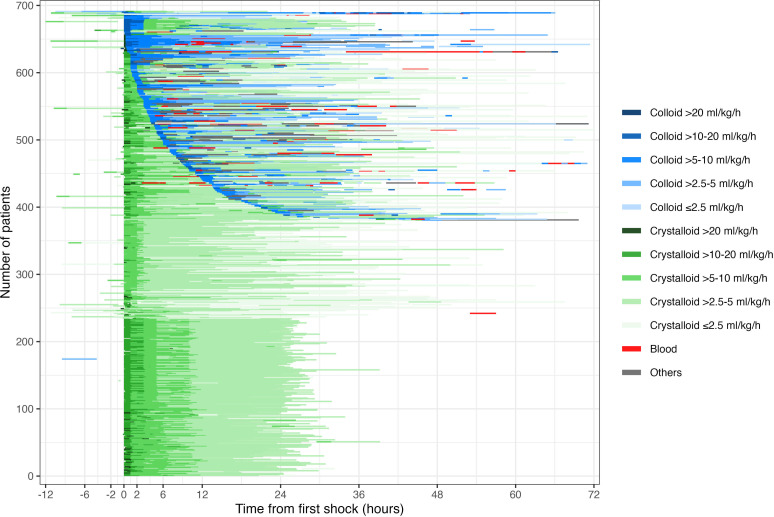
The vertical axis indicates the number of patients, with each line representing all fluids used for one patient. The blue colour represents colloid solutions, the green colour represents crystalloid solutions. The red colour represents blood. The degrees of colour lightness indicate how high the fluid rate per hour (mL/kg/hour) in which darker colour is for higher rate of fluid infusion. On the horizontal axis, zero is the time of onset of DSS. Blood includes whole blood and packed red cells, as well as blood products (plasma, cryoprecipitate, platelets, fresh frozen plasma and platelet-rich plasma) when used for bleeding or serious coagulation problems. Colloids include hydroxyethyl starch solutions, gelatin, albumin and plasma/platelet-rich plasma when used for volume expansion. Crystalloids include Ringer’s lactate, Hartmann’s Sterofundin, 0.9% saline and 5% dextrose in 0.9% saline. Other fluids include glucose 10%–30% with or without added electrolytes, nutritional support, etc. DSS, dengue shock syndrome.

Individual fluid management for all 691 children over the first 72 hours from shock presentation is shown in figure 1, demonstrating the wide range of fluid strategies employed in terms of fluid type, rate and duration. Although slightly more than half the patients received crystalloid fluids alone for the whole period, 308/691 (45%) received a colloid infusion, (either hydroxyethyl starch, gelatin or platelet-rich plasma, with 20% albumin prescribed in one case) at some point during this time. The latter group also received blood products more frequently. Some patients (25/691, 4%) received a continuous colloid infusion for more than 24 hours. Notable differences were apparent in the strategies adopted at different sites (online supplemental figure S5).

### Effects of initial resuscitation on outcomes

The total volume of parenteral fluid was comparable between the two groups over the first 24 hours but was slightly greater in the mixed-fluid than the crystalloid-only group by 72 hours (123 (IQR 96–143) vs 118 (IQR 85–131) mL/kg) (tables1  2, online supplemental figure S6).

**Table 2:** Unadjusted and adjusted results comparing outcomes between the two initial resuscitation groups

Outcome	Type of ES	Unadjusted analysis			Adjusted analysis		
		ES	95% CI	P value	ES	95% CI	P value
Time to initial shock recovery	HR	0.80	0.66 to 0.98	0.028	0.85	0.61 to 1.20	0.360
Reshock	OR	1.57	0.94 to 2.55	0.078	0.94	0.53 to 1.58	0.812
Time to first reshock	HR	1.54	0.98 to 2.40	0.060	0.94	0.57 to 1.56	0.819
Number of reshock episodes	RR	1.51	0.94 to 2.37	0.079	0.99	0.59 to 1.62	0.982
Respiratory compromise	OR	4.24	2.70 to 6.63	<0.001	**2.20**	**1.37 to3.48**	**<0.001**
Time to respiratory compromise	HR	3.72	2.57 to 5.37	<0.001	**2.08**	**1.11 to3.88**	**0.022**
Requirement for NCPAP or ventilation	OR	5.19	2.97 to 9.04	<0.001	**2.34**	**1.02 to5.37**	**0.045**
Severe bleeding	OR	1.65	0.64 to 3.80	0.263	0.57	0.18 to 1.80	0.338
Time to final shock recovery	HR	0.63	0.51 to 0.77	<0.001	0.51	0.24 to 1.07	0.075
Days in hospital after DSS onset	RR	1.28	1.18 to 1.40	<0.001	**1.33**	**1.03 to1.73**	**0.032**

The ‘crystalloid -only’ group is the reference group.

Bold values are statistically significant.

DSSdengue shock syndromeESeffect sizeNCPAPnasal continuous positive airway pressureRRrisk ratio

Although mortality rates were similarly low in both groups (2%), in the unadjusted analyses, other outcomes were generally more favourable in the crystalloid-only group. Reshock occurred less frequently and at a later time point in this group, but the differences were not statistically significant. The final shock recovery time was significantly shorter in the crystalloid-only group (11 (IQR 9–21) vs 19 (IQR 10–28) hours, HR 0.63, p<0.001), while respiratory compromise occurred more frequently (45% vs 15%) and at an earlier time point (10 vs 22 hours, HR 4.24, p<0.001), among the mixed-fluid recipients, with a significantly greater need for intervention (HR for either nasal continuous positive airway pressure (NCPAP) or mechanical ventilation of 5.19, p<0.001). Severe bleeding was also observed more frequently in the mixed-fluid group, but the difference was not significant.

After adjusting for baseline severity using the IPW score, the following outcomes remained significantly worse among mixed-fluid than among crystalloid-only recipients: respiratory compromise (HR 2.08, p=0.022); requirement for NCPAP or mechanical ventilation (OR 2.34, p<0.045); and days in hospital after DSS onset (RR 1.33, p=0.032). In online supplemental figure S6, Kaplan-Meier curves are shown for the major outcomes, highlighting in particular that respiratory compromise occurred more frequently and at an earlier time point among the mixed-fluid recipients.

### Effects of general management strategy on outcomes

**Figure 2:**
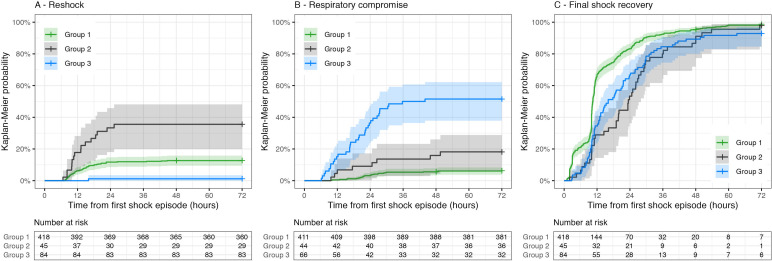
Group 1: conservative-colloid; group 2: intermediate-colloid; group 3: liberal-colloid. Note that for respiratory compromise, the number at risk excludes cases with missing data and cases with respiratory compromise within the first 6 hours from shock onset (ie, within the time frame of the overall management strategy definition).

Among the 547 cases included in this analysis, clear trends towards administration of greater total fluid volumes and greater colloid volumes at 24 and 72 hours were apparent across groups 1, 2 and 3 (tables1  3, figure 2).

**Table 3:** Unadjusted and adjusted results comparing outcomes between the three general management strategy groups

Outcome	Type of ES	Unadjusted analysis			Adjusted analysis		
		ES	95% CI	P value	ES	95% CI	P value
Reshock	OR						
Intermediate-colloid		3.80	1.90 to 7.40	<0.001	0.71	0.19 to 2.64	0.609
Liberal-colloid		0.08	0.00 to 0.39	0.014	0.14	0.02 to 1.07	0.058
Time to reshock	HR						
Intermediate-colloid		3.22	1.84 to 5.63	<0.001	0.73	0.21 to 2.53	0.618
Liberal-colloid		0.09	0.01 to 0.64	0.016	0.15	0.02 to 1.10	0.062
Number of reshock episodes	RR						
Intermediate-colloid		3.05	1.68 to 5.39	<0.001	0.95	0.27 to 3.32	0.939
Liberal-colloid		0.08	0.00 to 0.36	0.012	**0.13**	**0.02 to0.93**	**0.043**
Respiratory compromise	OR						
Intermediate-colloid		3.29	1.31 to 7.53	0.007	0.62	0.14 to 2.75	0.531
Liberal-colloid		15.73	8.49 to 29.75	<0.001	**8.84**	**4.27 to18.27**	**<0.001**
Time to respiratory compromise	HR						
Intermediate-colloid		3.05	1.38 to 6.74	0.006	0.62	0.15 to 2.60	0.516
Liberal-colloid		11.37	6.81 to 18.98	<0.001	**6.94**	**3.79 to12.70**	**<0.001**
Requirement for NCPAP or ventilation	OR						
Intermediate-colloid		1.57	0.24 to 6.02	0.561	0.11	0.01 to 1.15	0.065
Liberal-colloid		17.84	8.80 to 38.27	<0.001	**8.16**	**3.00 to22.17**	**<0.001**
Severe bleeding	OR						
Intermediate-colloid		1.06	0.06 to 5.82	0.959	0.15	0.01 to 1.63	0.120
Liberal-colloid		2.27	0.60 to 7.16	0.181	2.94	1.53 to 16.28	0.216
Time to final shock recovery	HR						
Intermediate-colloid		0.54	0.40 to 0.74	<0.001	**0.72**	**0.57 to0.91**	**0.005**
Liberal-colloid		0.58	0.45 to 0.74	<0.001	**0.73**	**0.56 to0.94**	**0.016**
Days in hospital after DSS onset	RR						
Intermediate-colloid		1.20	1.04 to 1.37	0.010	1.01	0.91 to 1.12	0.847
Liberal-colloid		1.31	1.18 to 1.45	<0.001	**1.15**	**1.03 to1.29**	**0.015**

Conservative-colloid is the reference group.

All adjusted estimates are derived from models using the inverse probability weighting score and robust sandwich-type variance estimators. Among the adjusted analyses, statistically significant results are indicated in bold typeface.

DSS, dengue shock syndrome; ES, effect size; NCPAP, nasal continuous positive airway pressure; RR, risk ratio

Notably, reshock occurred in only 1/84 (1%) patient in group 3 (liberal-colloid) compared with 53/418 (13%) in group 1 (conservative-colloid) and 16/45 (36%) in group 2 (intermediate-colloid), as per the inferred general management categories, defined according to fluid use among individuals who were apparently cardiovascularly stable after initial resuscitation. Similarly, no individual in group 3 experienced a second episode of reshock, compared with 2% in group 1 and 11% in group 2. When compared with group 1, the risk for reshock was higher among group 2 patients (OR 3.8, p<0.001) but lower among group 3 patients (OR 0.08, p=0.014).

Conversely, however, individuals in group 3 showed significantly worse outcomes for all respiratory parameters assessed: development of respiratory compromise; need for respiratory support; need for diuretic therapy. Other complications such as severe bleeding, severe liver impairment and acute kidney injury occurred infrequently, and very few children overall required inotropic support. Individuals in group 1 had the shortest time to achieve final recovery (10 hours (IQR (9–17)), but all three deaths occurred in this group. Meanwhile, final recovery times in group 2 and group 3 were 23 hours (IQR (11–29)) and 15 hours (IQR (10–26)), respectively.

Using the IPW score to adjust for baseline severity, the following outcomes remained significantly different for group 3 compared with group 1: the final shock recovery time was longer ingroup 3 (HR 0.73, p=0.016) and there were fewer reshock episodes (RR 0.13, p=0.043); development of respiratory compromise was more common (OR 8.84, p<0.001) and it occurred at an earlier time point (HR 6.94, p<0.001); the requirement for NCPAP or ventilation was greater (OR 8.16, p<0.001) and patients in this group spent more days in hospital after DSS onset (RR 1.15, p=0.015) than those in group 1. When comparing group 2 to group 1 and adjusting for baseline severity, only the final shock recovery time was significantly different (HR 0.72, p=0.005). The Kaplan-Meier curves presented in figure 2 illustrate differences in these major outcomes visually.

### Costs of different fluid strategies

Among the Vietnamese patients, estimated costs for the mixed-fluid initial resuscitation group were significantly higher than those for the crystalloid-only group, after adjustment for baseline severity. Considering the general management strategy, estimated costs for group 3 were significantly higher (almost triple) those for group 1, and twice those for group 2, with the estimates dominated by costs for the fluid solutions themselves and for emergency/ICU bed occupancy (online supplemental tables S1–S3).

## Discussion

Although WHO guidelines for dengue management have existed for many years and are widely disseminated, here we describe how the fluid strategies actually employed for paediatric DSS cases vary considerably across centres of excellence in dengue-endemic settings in Southeast Asia. The findings indicate that, among patients of similar baseline severity, use of mixed colloid/crystalloid regimens for initial fluid resuscitation may not provide better outcomes compared with crystalloids alone. Second, among children who appeared cardiovascularly stable immediately after the initial resuscitation, prolonged colloid use reduced the number of reshock episodes but was associated with increased risk for respiratory compromise and need for ventilatory support. Prolonged colloid use did not increase the risk of bleeding but did increase the length of hospital stay and the cost of management. These results suggest that prolonged use of colloid solutions, once initial cardiovascular instability has improved, may be inappropriate, especially in settings without access to respiratory support.

Historically, administration of synthetic colloids became an accepted strategy for management of hypovolaemic shock during the latter half of the 20th century. Dextran solutions were among the first available and their use by Thai physicians clearly improved mortality from DSS.^20^ HES solutions were introduced in the 1960s, and soon became the most commonly used synthetic colloids in western ICUs. Subsequently, however, multiple large trials in high-income countries (HICs) raised safety concerns about hydroxyethyl starch (HES) solutions,^1221^,^25^ resulting in the transition towards use of human albumin alongside early intervention with inotropic agents. The WHO DSS management guidelines continue to advocate the use of synthetic colloids for profound and recurrent shock, and many experienced clinicians, having not observed apparent adverse effects in their regular clinical practice, continue to use HES solutions in these circumstances. Dextrans and gelatins are also still used in countries where they remain available, although formal assessment of any associated adverse events is lacking since these solutions are now rarely deployed in the HICs where major fluid resuscitation studies have taken place.

Given that a transient systemic vascular leak syndrome underlies the hypovolaemic shock that is characteristic of DSS, synthetic colloids with an average molecular weight similar to albumin, the major protein constituent of human plasma (eg, dextrans) might be the preferred choice to replace the leaked proteins in cases where crystalloids alone prove ineffective. On the other hand, larger molecular weight molecules (eg, starch solutions) might theoretically remain for longer in the intravascular space and potentially reduce the risk for subsequent reshock.^26 27^ The rapid effect of colloid solutions in restoring cardiac index was first demonstrated in a pilot randomised fluid trial of DSS management in Vietnamese children almost 30 years ago.^28^ The two subsequent randomised trials, both single-centre and employing a conservative-colloid general strategy, showed no clear advantage in reducing the reshock rate or overall shock recovery time for colloids over crystalloids when used for initial DSS resuscitation.^8 10^ No differences were observed in bleeding manifestations, coagulation derangements, the severity of fluid overload or other complications.^10^ However, it is important to recognise that the adult studies in which HES-related adverse effects (renal compromise, increased need for blood transfusion) were identified were large, typically involving thousands rather than hundreds of study participants, and compared crystalloid/colloid use throughout the ICU stay rather than focusing on initial resuscitation.^21 22 25^ On the other hand, it is also important to acknowledge that DSS occurs primarily in children and young adults, in whom cardiovascular health is likely to be excellent and chronic diseases or comorbidities are rare; the potential for adverse consequences of colloid use may be different in this population, and the results of research in older adults should not simply be extrapolated to the young.

In addition to the type of fluid, controversy also exists around the volume and rate of administration for shock resuscitation. For dengue, there is evidence indicating the risk for respiratory distress increases significantly as the infused fluid volume over the preceding 24 hours increases.^29^ Contrary to expectations, mortality in African children with severe febrile illness increased after resuscitation with fluid boluses.^30^ Rapid correction of hypovolaemia has traditionally been accepted as an important feature of shock resuscitation, but in recent years increasing evidence suggests that aggressive fluid replacement may be harmful to the microvasculature.^31^ In this retrospective study, we were unable to explore potential effects related to fluid volume and rate of administration. However, it is clear that, like any therapeutic intervention, parenteral fluid therapy has both beneficial and adverse effects, and these vary depending on the clinical context.

The economic burden of dengue in Vietnam, as in most dengue-endemic settings, is significant, with medical costs consistently making the largest contribution to overall costs.^9^ Here, we present the first report that specifically focuses on DSS management, demonstrating that fluid costs and bed costs are major contributing factors. In Vietnam, DSS patients are always managed in ICU/HDU settings to facilitate the frequent monitoring required during the period of ongoing plasma leakage. Provision of respiratory support is clearly expensive, but the actual number of children requiring such support was small and thus the effect on overall costs was marginal. At the time of this study, albumin was essentially never used for children with DSS, due to restricted availability and prohibitive cost; HES solutions (around US$6/500 mL) were usually used when management with Lactated Ringer’s (around US$0.4/500 mL) was deemed insufficient. Notwithstanding potential benefits for individual children, adopting a general management strategy that included prolonged use of colloids did not improve overall outcomes but did increase the economic burden per patient significantly. In future research, this area needs to be carefully explored in order to better discriminate the effects of different fluid strategies to provide improved outcomes but also to ensure feasibility of application in LMIC settings.

The major limitation of the study is its retrospective nature. Collecting data from hospitals with different fluid management and monitoring protocols inevitably raises concerns about the completeness of the dataset. However, the assessment of data quality showed that for most important variables, the amount of missing information was actually small. We defined all the outcomes of interest before beginning the data analysis and used electronic algorithms to derive them, aiming to reduce personal bias. Additionally, by using the IPW score, we tried to adjust for any lack of equivalence between patients in the treatment groups. However, in addition to differences in baseline severity, other confounders related to institutional differences in approach to fluid use, blood product use and patient management/monitoring may have affected the analyses. In a retrospective study like this, uncertainty must remain about the link between patient severity and the fluid regimen chosen by the treating clinician, and about how this linkage could affect the outcomes of interest.

## Conclusions

In summary, fluid strategies for paediatric DSS management vary considerably between hospitals and countries in Southeast Asia. Initial colloid use may not provide better overall outcomes than crystalloids alone, and while prolonged colloid treatment did reduce the occurrence of reshock considerably, complications also occurred more frequently in this group, and management costs were markedly higher for colloid recipients. Many dengue experts still feel that there is a place for colloid use in children who respond poorly to initial volume replacement or decompensate repeatedly, but to date, there have been no formal research studies to explore this issue. At present, the choice of colloid is often determined by market forces, especially since the controversy around the use of HES solutions for adult shock resuscitation has limited supply in many countries.^32^,^35^ Continuous re-evaluation of the principles and practice of DSS management is important but should be driven by research evidence rather than market forces. A prospective systematic approach is needed, ideally incorporating a suitably powered large-scale randomised trial, but this analysis provides valuable initial insights into potential effects of fluid management strategies on DSS outcomes.

## supplementary material

10.1136/bmjgh-2024-017538 online supplemental figure 1

10.1136/bmjgh-2024-017538 online supplemental file 1

## Data Availability

Data are available on reasonable request.
