# Supplementary figures and images for: The influence of fluid resuscitation strategy on outcomes from dengue shock syndrome: a review of the management of 691 children in 7 Southeast Asian hospitals
Source: BMJ Glob Health. 2025 Mar 11;10(3):e017538. doi: 10.1136/bmjgh-2024-017538 (PMC11904338; doi:10.1136/bmjgh-2024-017538)

**Supplemental Figure S1: Study flowchart**

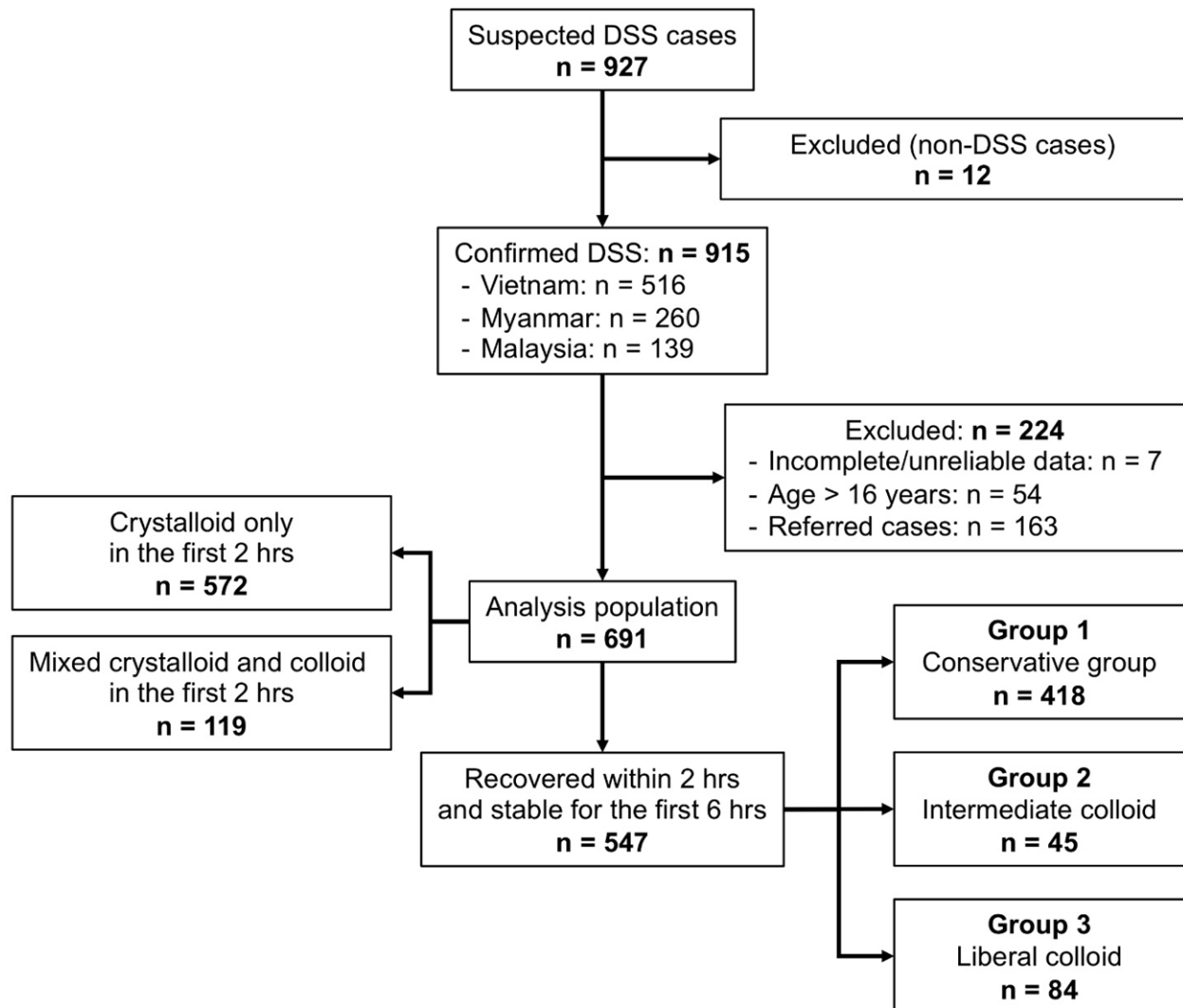

DSS: dengue shock syndrome

Supplement: online supplemental figure 1 [file bmjgh-10-3-s001.pdf]
