# Supplementary material for: The influence of fluid resuscitation strategy on outcomes from dengue shock syndrome: a review of the management of 691 children in 7 Southeast Asian hospitals
Source: BMJ Glob Health. 2025 Mar 11;10(3):e017538. doi: 10.1136/bmjgh-2024-017538 (PMC11904338; doi:10.1136/bmjgh-2024-017538)
Supplement: online supplemental file 1 [file bmjgh-10-3-s002.pdf]

Supplementary Appendix

Contents

Appendix S1. Outcome definitions 2

Appendix S2. Economic evaluation 3

Table S1. Costs for all fluids, procedures and medications used for DSS management at the Vietnam study sites 4

Table S2. Associations between the costs of different fluid strategies, after adjustment for baseline severity . 6

Table S3. Comparison of the costs of the different fluid strategies used to treat Vietnamese children with DSS in USD (2017 prices) 7

Appendix S3. Data quality assessment 8

Figure S2. Data availability for baseline characteristics at presentation with shock for the 691 DSS patients included in the analysis 10

Figure S3. Missing data for heart rate, blood pressure and respiratory rate during the first 24 hours from shock onset 11

Figure S4. Missing data for the main outcomes of interest among the 691 patients included in the final analysis 12

Figure S5. Overview of fluid use among children managed at three major centres that each contributed substantial case numbers 13

Figure S6. Kaplan-Meier curves displaying the estimated probability for A) development of reshock, B) development of respiratory compromise and C) final shock recovery, according to the initial resuscitation strategy 14

References 15

Appendix S1. Outcome definitions

1. Reshock was defined as an episode of cardiovascular decompensation recorded by the attending clinician in the hospital file, that occurred at least 6 hours after stabilization following the previous episode and resulted in a change in management.
2. Initial shock recovery time was defined as the time from dengue shock syndrome (DSS) onset to first achieving cardiovascular stability. Criteria of stable vital signs (pulse pressure [PP] ≥ 25mmHg and systolic blood pressure [SBP] ≥ 80 [age ≤ 5] or SBP ≥ 90 [age > 5]) for at least 6 hours, without use of inotropes. A single low measurement (PP < 25 or low SBP) was accepted within the 6 hour period provided there was no change in the intravenous fluid regimen used.
3. Final shock recovery time was defined as the time from DSS onset to achieving sustained cardiovascular stability – i.e., stable vital signs (PP, SBP limits as above) without subsequent cardiovascular deterioration, with an intravenous infusion fluid speed ≤4 mL/kg/h at this time and subsequently, and with no inotropes or whole blood or packed red cells given after this time.
4. Respiratory distress was defined as one or more of the following signs recorded in the file – shallow breathing, retractions, grunting, nasal flaring, accessory muscle use – usually with an increased respiratory rate for age.
5. Respiratory compromise was defined as any of the following: a) one or more signs of respiratory distress recorded in the file together with an increase in nasal oxygen flow; or b) need for respiratory support with nasal continuous positive airway pressure (NCPAP) or c) need for mechanical ventilation.
6. Acute kidney injury was defined if any creatinine level was more than 1.5 times the upper limit of normal (ULN) for age. ULN for age: ≤1 year: 35 umol/L; 1-2 years: 44 umol/L; 3-10 years: 61 umol/L; 11-16 years: 79 umol/L. Premorbid creatinine values were not available.
7. Severe liver impairment was defined as any AST or ALT level ≥ 1000 IU/L. Premorbid liver enzyme values were not available.
8. Severe bleeding was defined as any bleeding during the illness course which resulted in shock, and/or necessitated a blood transfusion or an intervention to stop bleeding (e.g., nasal packing), and/or involved a critical organ (brain, spinal cord), and/or resulted in death.
9. Hospital days after DSS onset was defined as discharge day minus day of DSS onset, plus 1.

Appendix S2. Economic evaluation

To assess the relative costs of the different fluid management strategies, we focused only on Vietnam as the costs of the various interventions were the same at the different study sites. For each patient we included all expenditure for bed costs, routine laboratory tests, intravenous fluids, blood products, respiratory support and antibiotic therapy, using the Vietnamese MoH pricelist 02/2017/TT-BYT issued in March 2017. Costs were first summarised in Vietnamese Dong and then converted to USD (**Table S1**).

With respect to bed costs, the MoH recommended charge for an intensive care unit (ICU) bed is greater than that for an emergency bed. For patients requiring nasal CPAP or mechanical ventilation we allocated a daily ICU bed charge for any day when such support was required, while for all other days during their hospitalization we allocated a daily emergency bed charge. For all other patients (i.e., those who never required nasal CPAP or mechanical ventilation) we allocated a daily emergency bed charge for the whole stay.

We were not able to do full economic costing as we did not have information on the costs for nursing services, physician services, extra laboratory tests, catering etc., nor on any additional costs incurred by the family, e.g., due to lost income/reduced productivity of the parents during their child's illness episode.

In the unadjusted analyses (**Table 5**, main report), considering the immediate response strategy total costs for children in the mixed-fluid group were higher than costs in the crystalloid-only group. Similarly, for the overall management strategy, total costs for groups 2 and 3 (i.e., those who received a colloid for some period of time during the first 6 hours) were significantly greater than for the crystalloid-only recipients. The majority of the excess costs were for fluid costs and bed charges.

Results for the comparisons done after adjusting for baseline severity using the IPW score are shown in **Table S2**. For the immediate response fluid strategy, the estimated cost for those in the mixed-fluid group remained significantly greater compared to the crystalloid-only group (R squared 0.398, $p < 0.001$). For the general management strategy analysis, Group 2 and Group 3 also had higher expenditure compare to Group 1 (R squared 0.271, $p < 0.001$).

Table S1. Costs for all fluids, procedures and medications used for DSS management at the Vietnam study sites

	Dosing Form	Price in USD*
**Resuscitation fluids**		
Lactated Ringer's	500 ml	0.4
Dextrose Saline	500 ml	0.4
Sodium chloride 0.9%	500 ml	0.3
Sodium chloride 3%	100 ml	0.3
Albumin 20%	100 ml	73.8
Glucose 5%	500 ml	0.3
Glucose 10%	250 ml	0.4
Glucose 30%	250 ml	0.5
Refortan 6% 200/0.5	500 ml	6.2
Gelofusin	500 ml	4.7
Voluven 6% 130/0.5	500 ml	8.3
**Blood products**		
PRBC from 350ml whole blood	175 ml	43.6
Whole blood	250 ml	38.8
Platelet concentrate – 6 units	320 ml	176.5
Cryoprecipitate	50 ml	16.0
Fresh frozen plasma	150 ml	7.9
**Sedation drugs**		
Midazolam	5 mg	0.7
Fentanyl	100 ug	0.4
**Antibiotics**		
**Oral Antibiotics**		
Amoxicillin/clavulanic acid	1200 mg	1.9
Amoxicillin	1500 mg	3.0
**IV Antibiotics**		
Cefotaxime	1000 mg	2.8
Cefepime	1000 mg	0.9
Ceftriaxone	1000 mg	8.1
Ceftazidime	1000 mg	3.4
Colistin	1 MU	16.0
Ciprofloxacin	500 mg	11.0
Clindamycin	600 mg	4.7
Oxacillin	1000 mg	0.7
Cefoperazone/sulbactam	1000 mg	2.1
Gentamicin	80 mg	0.1
Acyclovir IV	250 mg	15.6
Cloxacillin	1000 mg	0.7
Meropenem	500 mg	20.8
Metronidazole	500 mg	0.4
Ampicillin/sulbactam	1500 mg	3.0
Imipenem	500 mg	15.0
Levofloxacin	500 mg	5.5
Tazocin	4.5 g	10.0
Vancomycin	1000 mg	4.7
Amikacin	500 mg	3.4
**Inotropes**		
Adrenaline	1 mg	0.3
Dobutamine	250 mg	2.6
Dopamine	200 mg	0.9
Noradrenaline	4 mg	3.1
**Support for complications**		
Nasal prong oxygen	1 hour	0.1
Mask oxygen	1 hour	0.1
NCPAP	1 day	23.8
Mechanical ventilation	1 day	23.8
Intubation	1 time	24.8

	Dosing Form	Price in USD*
Arterial line procedure	1 time	6.0
Hemofiltration	1 time	282.4
Duosol/Hemosol	1 bag	29.1
**ICU bed**	1 day	28.3
**Emergency bed**	1 day	15.0
**Laboratory test**		
Full blood count		2.0
Bedside haematocrit		0.8
Dextrostix		1.0
AST		0.9
ALT		0.9
BUN		0.9
Creatinine		0.9
Electrolytes (sodium, potassium, chloride)		1.3
Albumin		0.9
Blood gas		9.5
Arterial lactate		4.3
Coagulation (APTT, PT, Fibrinogen, TT)		10.7
Dengue NS1		5.6
Ultrasound		2.2
Chest Xray		3.1

* 1 USD = 22370 VND (exchange rate in 2017)

ALT, alanine aminotransferase; APTT, activated partial thromboplastin time; AST, aspartate aminotransferase; BUN, blood urea nitrogen; DSS, dengue shock syndrome; ICU, intensive care unit; IV, intravenous; NCPAP, nasal continuous positive airway pressure; PRBC, packed red blood cell; PT, prothrombin time

Table S2. Associations between the costs of different fluid strategies, after adjustment for baseline severity

	Estimated value	P-value	Adjusted R-squared	P-value*
Initial resuscitation strategy			0.398	<0.001
Intercept	111.1			
Crystalloid only	0			
Mixed fluid	59.0	<0.001		
General management strategy				
Intercept	99.9		0.271	<0.001
Conservative-colloid	0			
Intermediate-colloid	59.7	0.09		
Liberal-colloid	141.1	<0.001		

All figures are in USD (2017 prices).

The ‘crystalloid only’ and conservative-colloid groups are the reference groups.

*: Multiple linear regression, after adjustment for baseline severity using the inverse probability weighting score

Table S3. Comparison of the costs of the different fluid strategies used to treat Vietnamese children with DSS in USD (2017 prices)

	Initial resuscitation			General management strategy			
	**Crystalloid only (N=289)**	**Mixed fluid (N=62)**	**p-value**	**Conservative-colloid (Group 1, N=258)**	**Intermediate-colloid (Group 2, N=26)**	**Liberal-colloid (Group 3, N=66)**	**p-value**
Laboratory costs	11.2 (7.9-15.8)	19.5 (12.6-32.4)	<0.001	10.1 (7.8 15.5)	13.8 (10.0-17.0)	23.6 (14.3-34.3)	<0.001
Fluid costs	3.5 (2.4-26.4)	63.8 (43.9-122.7)	<0.001	3.5 (2.4-4.6)	45.6 (28.1.0-63.8)	87.2 (55.9-122.6)	<0.001
Respiratory support*	8.0 (6.4-9.7)	8.0 (6.4-41.7)	0.009	8.0 (6.4-9.7)	6.4 (6.4-9.7)	9.7 (6.4-47.7)	<0.001
Medication costs[&]	25.3 (8.1-42.2)	33.1 (9.6–98.9)	0.642	25.3 (8.1-42.0)	NA	33.1 (12.6-43.8)	NA
Bed costs	75.1 (60.1-90.1)	75.1 (60.1-116.6)	0.011	75.1 (60.1-90.1)	60.1 (60.1-90.1)	90.1 (60.1-119.2)	0.004
**Total costs**	106.2 (77.9-137.6)	182.8 (136.0-277.2)	<0.001	98.7 (77.3-130.5)	137.3 (120.5-171.1)	223.5 (156.9-319.4)	<0.001

Summary statistic is median (interquartile range). All statistical tests are non-parametric.

Fluid costs include the costs for all colloids, crystalloids, blood and blood products used.

*: Includes costs for oxygen, NCPAP, ventilation. All patients are included here since nasal oxygen is mandatory for children with DSS according to national guidelines

[&]: Includes costs of inotropes, sedation, antibiotics and hemofiltration, for patients who received any of these interventions. (n=23)

DSS: dengue shock syndrome; NA: not applicable; NCPAP: nasal continuous positive airway pressure

Appendix S3. Data quality assessment

Baseline data: Data were available for most baseline characteristics apart from information on respiratory rate, respiratory distress and the results of confirmatory dengue diagnostics (**Figure S1**).

Patients with dengue shock are typically alert and, unlike those with septic shock, rarely have overt signs of respiratory distress at presentation; thus the focus of clinical staff in busy centres (where 5-10 new DSS cases may present each day during the season) is directed toward recording cardiovascular parameters rather than respiratory rate. Data were missing on blood pressure in a small number of cases at presentation, mainly due to the patients being profoundly hypotensive on arrival with no measurable blood pressure. Information on presence/absence of respiratory distress was missing in 48 cases, but none of these patients received any respiratory support during their hospital stay, and all recovered and were discharged home.

In dengue-endemic countries, the diagnosis of DSS is usually made clinically, when patients arrive at hospital with a typical history, symptoms and signs. Diagnostic tests are frequently not available (especially during epidemics), may be expensive (with the cost generally borne by the family), and tend to be used primarily for atypical DSS cases when clinicians are considering alternative diagnoses. A 10-year prospective study at one hospital in Vietnam previously demonstrated that among unselected cases undergoing laboratory diagnostic testing, a clinical diagnosis of DSS was correct in 95% of cases,[1] i.e., similar to the confirmation rate of 94% found among the sub-set of patients in this analysis for whom diagnostic test results were available.

Haematocrit measurements are an essential part of the assessment of DSS cases, as they reflect the severity of haemoconcentration and are routinely used to assess the effects of fluid therapy. Meanwhile, platelet measurements are often thought of in terms of the risk for severe bleeding, which is uncommon in children.[2] Thus baseline haematocrit values were almost invariably recorded (only 3 missing values) while platelet values were missing from the files in 33 cases.

Subsequent vital signs data: The proportion of missing data during the first 24 hours of observation after presentation with shock is shown in **Figure S2**, summarized in 2 hourly intervals (-1/+1) for pulse and blood pressure and 4 hourly intervals (-2/+2) for respiratory rate. These intervals were selected as being broadly representative of clinical practice across the 3 countries. Although in many centres the recommendation is for hourly observations for all DSS cases until cardiovascular stability is achieved, in practice the capacity for frequent monitoring is determined by workload and staff availability. Therefore, depending on the monitoring capacity at each study centre, the time interval for pulse and blood pressure measurement was every 1-4 hours, while the time interval for respiratory rate monitoring was typically every 3-4 hours.

As shown in the figure, heart rate and blood pressure values were recorded at least every 2 hours in around 90% of cases initially, falling to around 80% of cases from 6 hours after shock onset, remaining around that level for the rest of the 24 hour period. Respiratory rates were recorded less frequently, and were missing in around 20% of cases during each 4 hour interval.

Outcome data: Overall, the amount of missing data for the outcomes of interest was small, apart from for severe liver impairment and acute kidney injury.

In addition to death, major outcomes such as development of reshock, respiratory distress or severe bleeding are easily recognized by experienced clinicians and considered important enough to be clearly recorded in the hospital file. However, more than 50% of the information on acute kidney injury and severe liver impairment is missing. In children, the most common complication of dengue infection is severe plasma leakage progressing to hypovolaemic shock and organ impairment is unusual, except in severe cases with profound or prolonged shock. Therefore, in many situations, clinicians do not routinely check renal or liver function. Liver transaminase and creatinine are tested in complex cases with decompensated shock, prolonged shock or repeated reshock, which explains the high proportion of missing data on acute kidney injury and severe liver impairment.

To derive the outcome "final shock recovery time", a number of criteria must be satisfied – survival, normal SBP for age, PP ≥ 25 mmHg, no reshock, no blood transfusion, no inotrope use, and fluid speed ≤ 4 ml/kg/hour after

that time point. For 29 patients (4%) it was not possible to satisfy all these criteria and thus this outcome is missing. Aside from the 11 deaths, in many cases this was because very low dose inotropes or parenteral fluid therapy were maintained to the end of the 72-hour data collection period, often during the night pending clinical review on morning rounds.

Figure S2. Data availability for baseline characteristics at presentation with shock for the 691 DSS patients included in the analysis

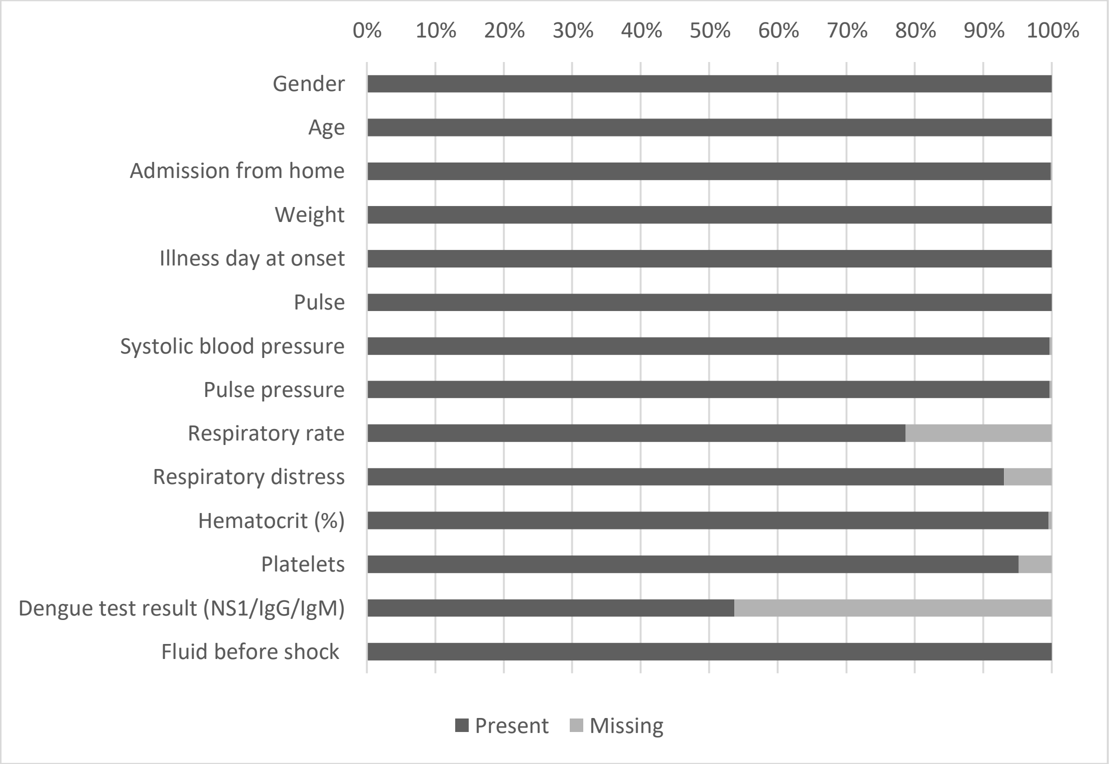

Figure S3. Missing data for heart rate, blood pressure and respiratory rate during the first 24 hours from shock onset

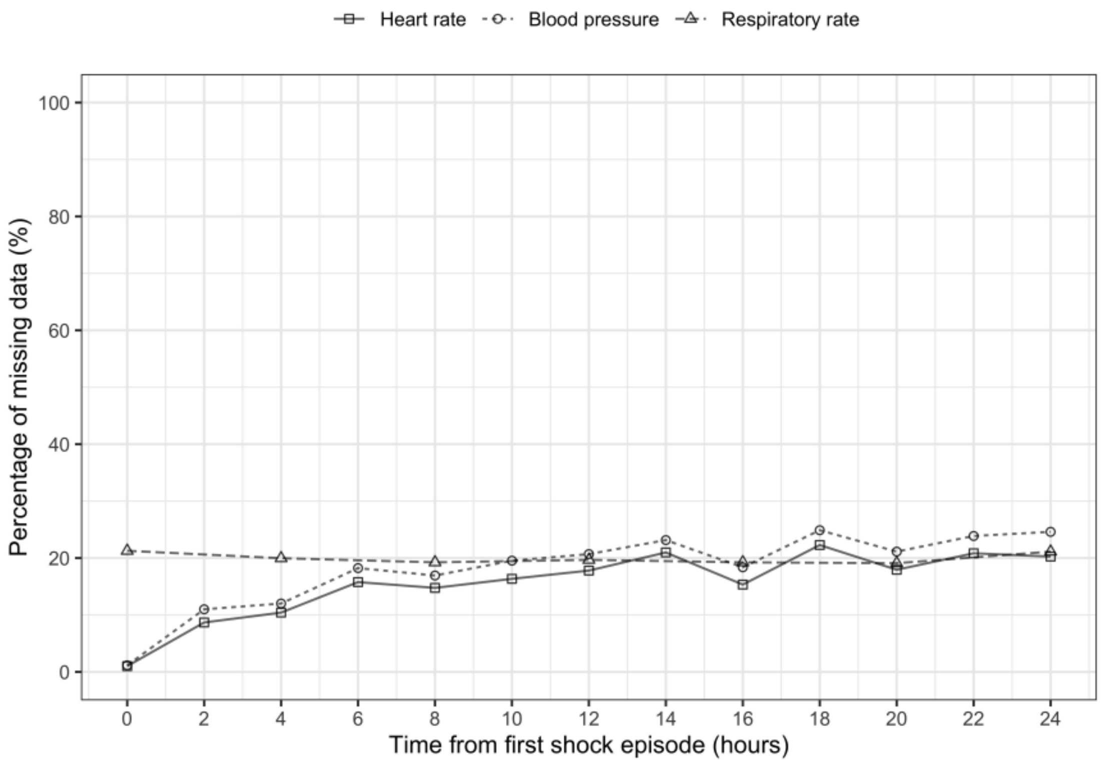

Figure S4. Missing data for the main outcomes of interest among the 691 patients included in the final analysis

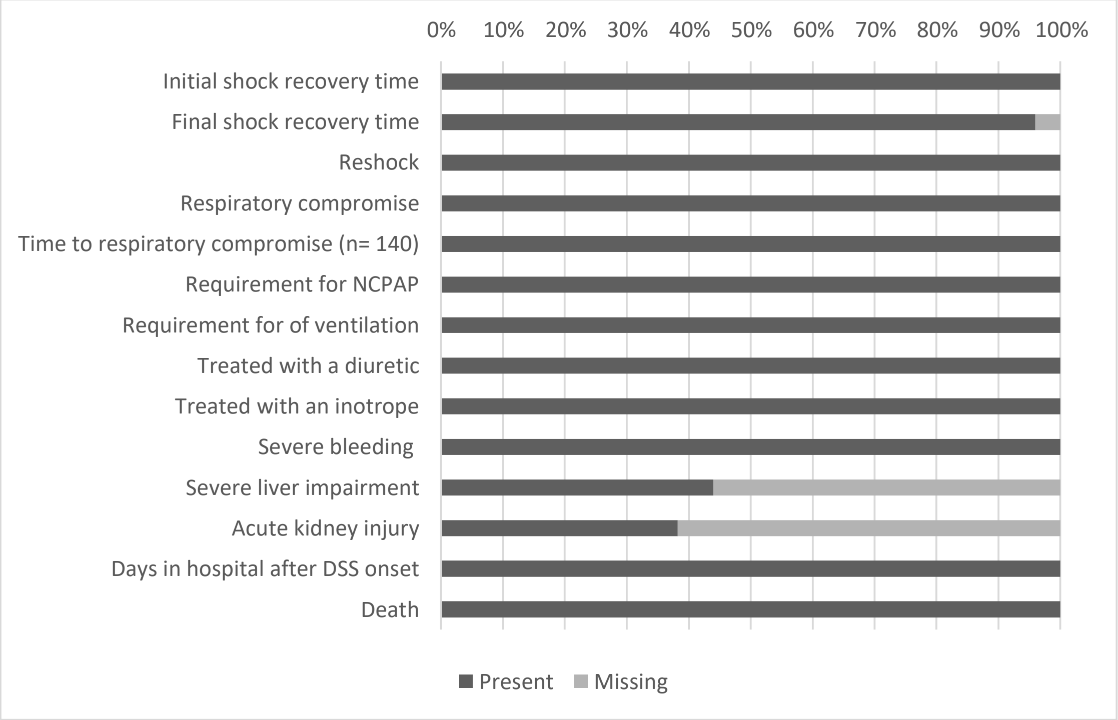

Figure S5. Overview of fluid use among children managed at three major centres that each contributed substantial case numbers

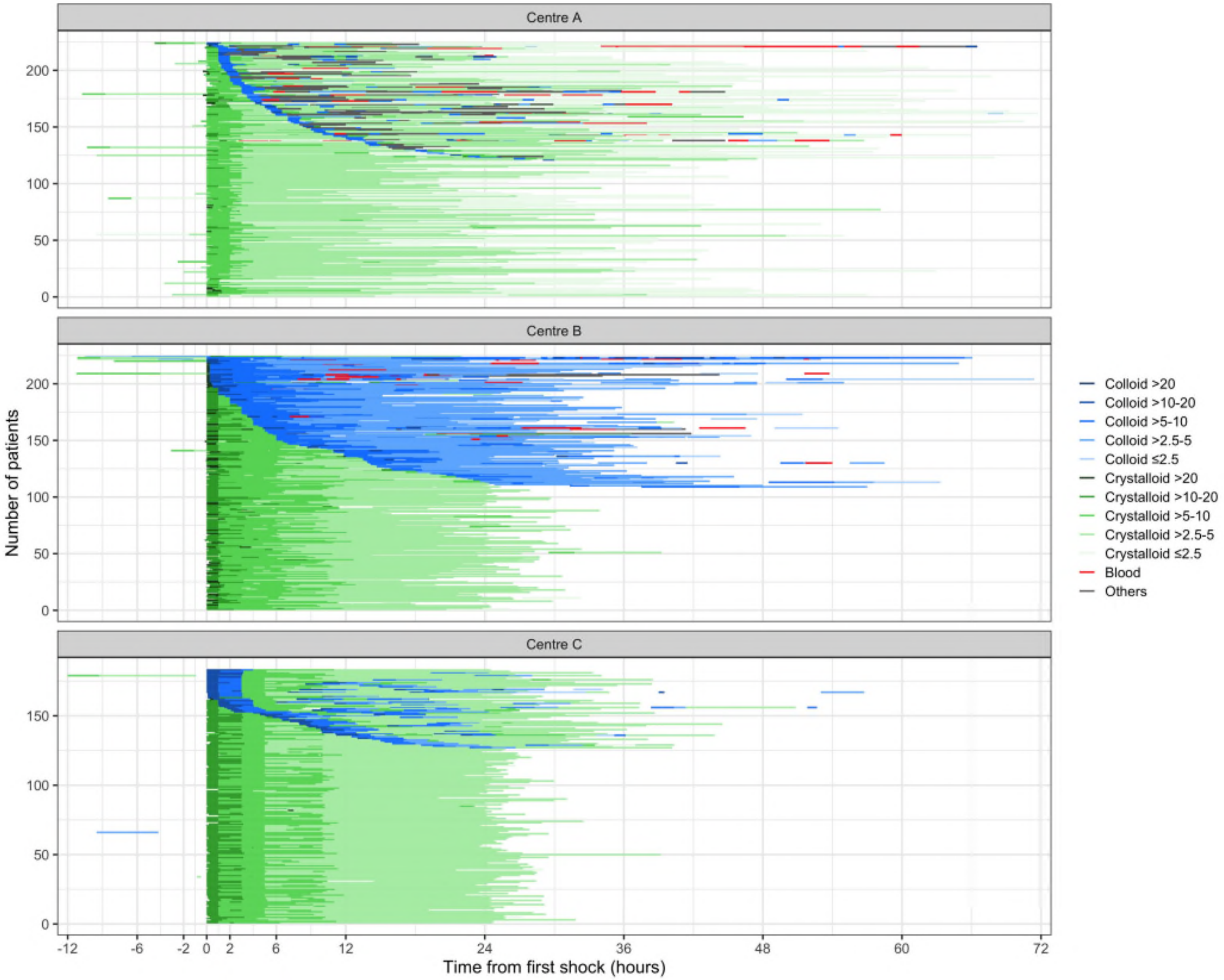

For each centre, the vertical axis indicates the number of patients managed there, with each line representing all the fluid used for one patient. On the horizontal axis, time=0 is the time of onset of DSS. Blood includes whole blood and packed red cells, as well as blood products (plasma, cryoprecipitate, platelets, fresh frozen plasma, and platelet-rich plasma) when used for bleeding or serious coagulation problems. Colloids include hydroxyethyl starch solutions, gelatin, albumin and plasma/platelet-rich plasma when used for volume expansion. Crystalloids include Ringer's Lactate, Hartmann's, Sterofundin, 0.9% saline, and 5% dextrose in 0.9% saline. Other fluids include glucose 10-30% with or without added electrolytes, nutritional support, etc.

Figure S6. Kaplan-Meier curves displaying the estimated probability for A) development of reshock, B) development of respiratory compromise and C) final shock recovery, according to the initial resuscitation strategy

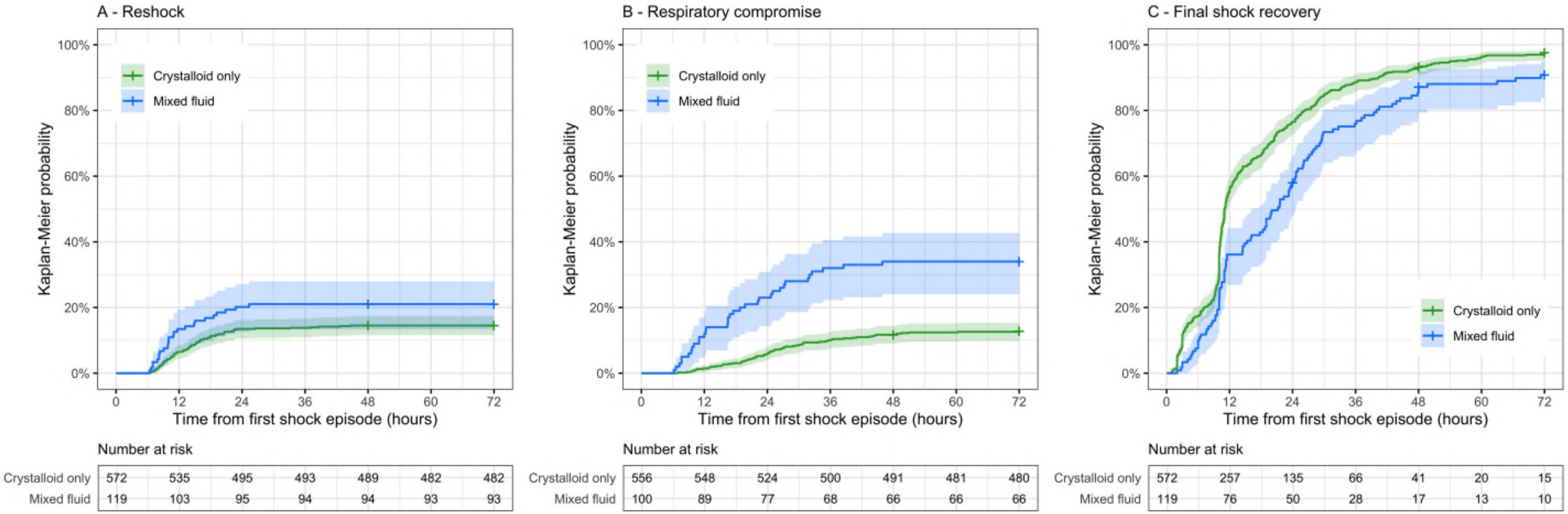

Note that for respiratory compromise, the number at risk excludes cases with missing data and cases with respiratory compromise within the first 2 hours from shock onset (i.e., within the time-frame used to categorise the initial resuscitation strategy)

References

1. Lam PK, Tam DT, Diet TV, Tam CT, Tien NT, Kieu NT, et al. Clinical characteristics of Dengue shock syndrome in Vietnamese children: a 10-year prospective study in a single hospital. Clin Infect Dis. 2013;57(11):1577-86.

2. Trung DT, Thao le TT, Dung NM, Ngoc TV, Hien TT, Chau NV, et al. Clinical features of dengue in a large Vietnamese cohort: intrinsically lower platelet counts and greater risk for bleeding in adults than children. PLoS Negl Trop Dis. 2012;6(6):e1679.
